# Supplementary material for: Potential and functional prediction of six circular RNAs as diagnostic markers for colorectal cancer
Source: PeerJ. 2022 May 19;10:e13420. doi: 10.7717/peerj.13420 (PMC9124462; doi:10.7717/peerj.13420)
Supplement: Supplemental Information 1 [file peerj-10-13420-s001.pdf]

Figure S1

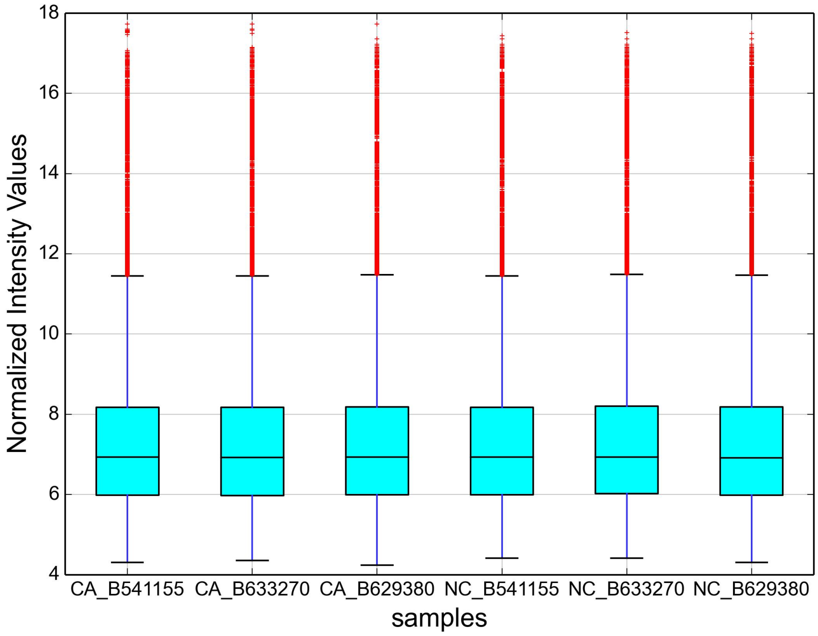

Figure S1 Boxplot represents the data quality of each chip is reliable after data standardization

Figure S2

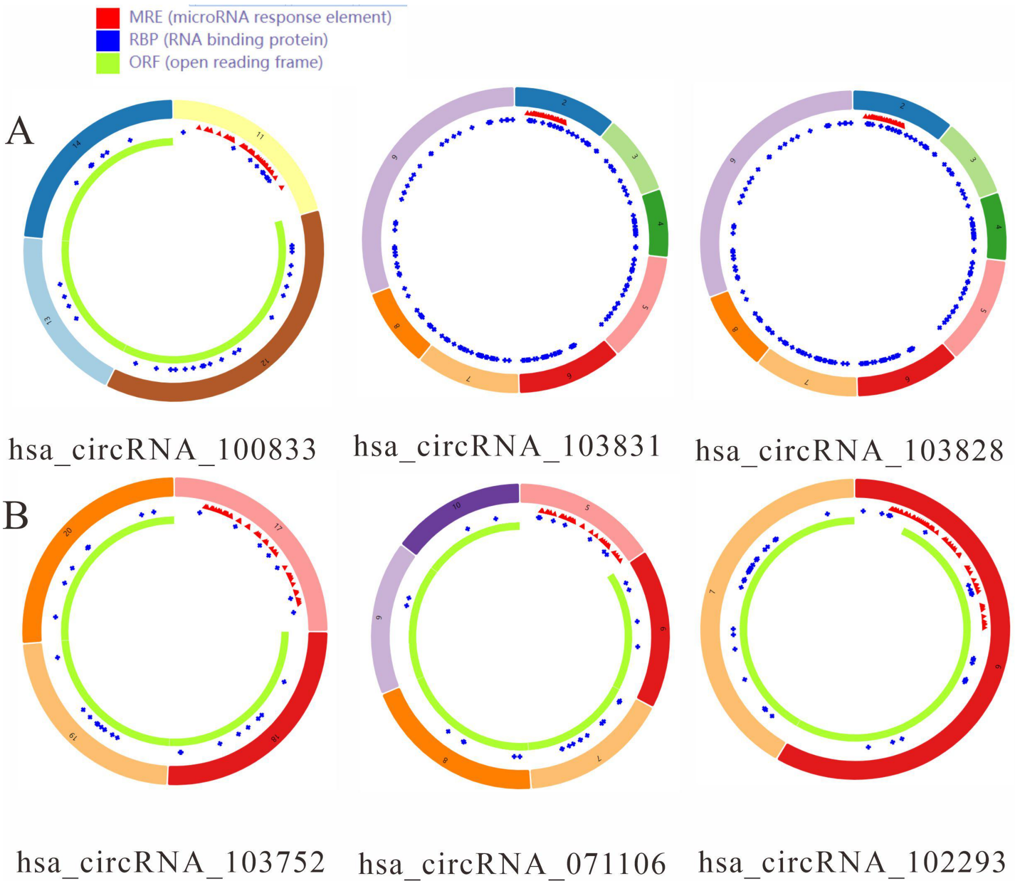

Figure S2 The basic structural patterns of 3 up-regulated circRNAs and 3 down-regulated circRNAs predicted by CSCD. (A) hsa_circRNA_100833, hsa_circRNA_103831, hsa_circRNA_103828. (B) hsa_circRNA_103752, hsa_circRNA_071106, hsa_circRNA_102293. MicroRNA response element (MRE) is represented in red. The RNA binding protein (RBP) is represented in blue. The open reading frame (ORF) is represented in green.

Figure S3

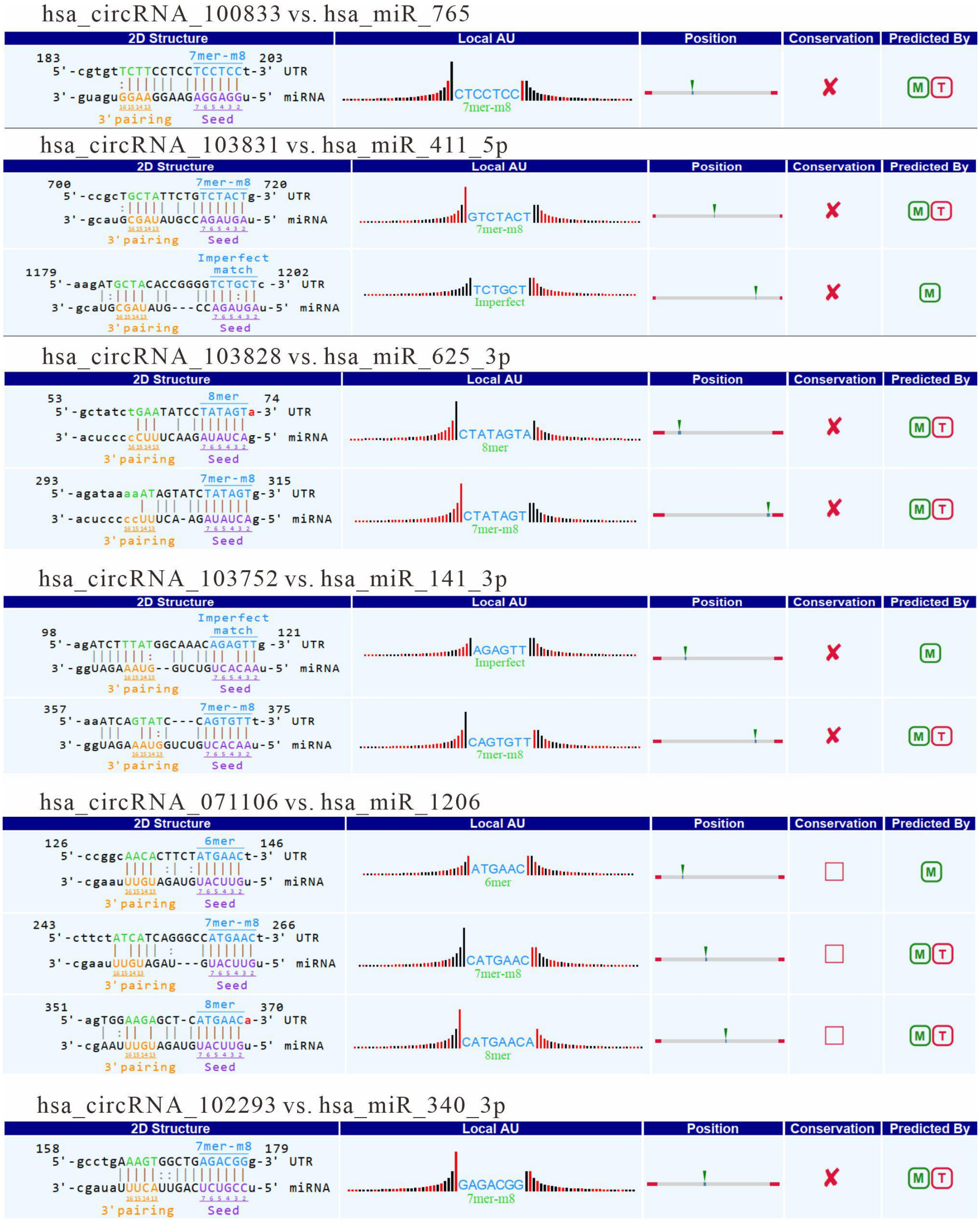

Figure S3 The details of circRNA-miRNA potential interaction sites. MiRNA Binding Sites, circRNA and miRNA Binding secondary structure.

Figure S4

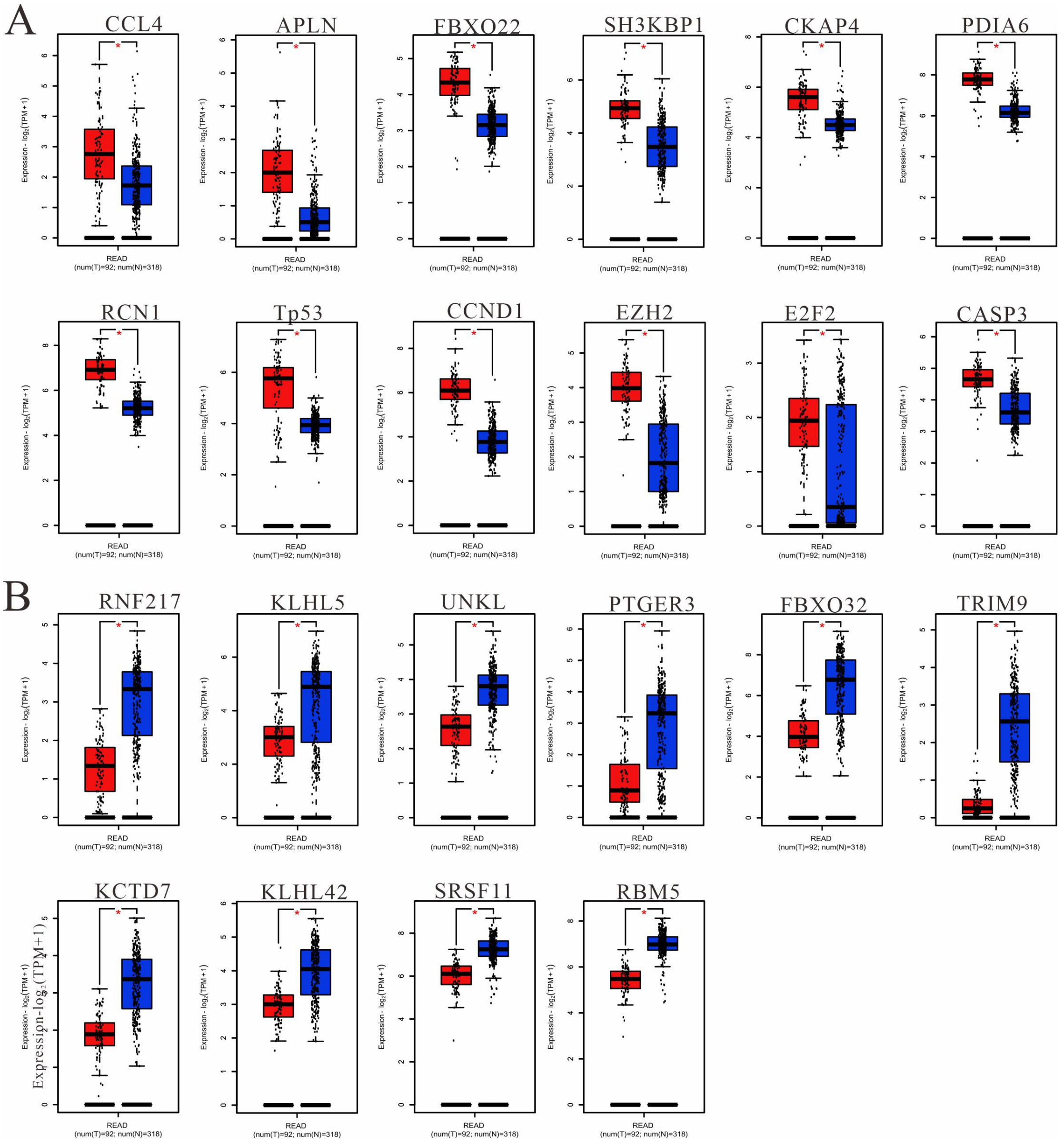

Figure S4 Hub gene expression. (A) Up-regulated hub genes via GEPIA analysis in READ tissues. (B) Down-regulated hub genes via GEPIA analysis in READ tissues. "*" represent $P<0.05$, $P<0.05$ indicated a difference in comparison between the two groups.

Figure S5

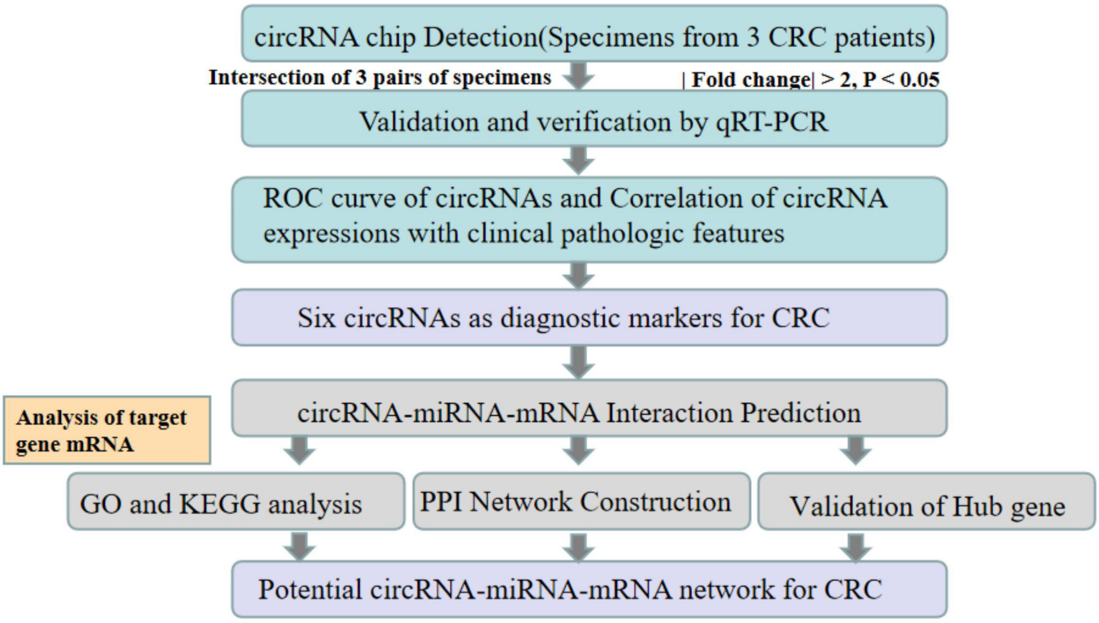

Figure S5 Flow Chart of this study
