# Supplementary material for: Potential and functional prediction of six circular RNAs as diagnostic markers for colorectal cancer
Source: PeerJ. 2022 May 19;10:e13420. doi: 10.7717/peerj.13420 (PMC9124462; doi:10.7717/peerj.13420)
Supplement: Supplemental Information 4 [file peerj-10-13420-s004.docx]

**Table S1.** Primer sequences are used for qRT-PCR.

| **Gene symbol** | **Primer sequence** |
| --- | --- |
| hsa_circRNA_100833 | ATGCTGCACGTGTTTGTTCT |
|  | GAACTTGCCCACGAATTCCA |
| hsa_circRNA_103831 | GCTCGGATGTTGCTGAATGA |
|  | TCCCACGAAGCCCCAAAG |
| hsa_circRNA_103828 | TGAAGGAGTAGGACTTGTGCA |
|  | CTTTTGGCCAGCAAGCTTCT |
| hsa_circRNA_103752 | TGCCCACCAACTTCAGAGAT |
|  | CCATTTTCCATGCAGCCCTT |
| hsa_circRNA_071106 | AGGCATACTGACTCCATTGACA |
|  | TTTTCTTCCGATCAGCAGCT |
| hsa_circRNA_102293 | AAACGAAACTCTCCGACAGC |
|  | AACTCATCCTCTTTCAGTCTCTC |
