# Supplementary material for: Potential and functional prediction of six circular RNAs as diagnostic markers for colorectal cancer
Source: PeerJ. 2022 May 19;10:e13420. doi: 10.7717/peerj.13420 (PMC9124462; doi:10.7717/peerj.13420)
Supplement: Supplemental Information 5 [file peerj-10-13420-s005.docx]

**Table S2.**Top 50 hub genes in the PPI network in CRC.

| **circRNA** | **miRNA** | **mRNA** |
| --- | --- | --- |
| hsa_circRNA_100833 | hsa-miR-765  hsa-miR-495-3p  hsa-miR-665  hsa-miR-193b-5p  hsa-miR-124-5p | HECTD2 AREL1 UBE2H LTN1 UFL1 UBE2W ASB11 ASB15 KLHL42 RNF41 KLHL5 KBTBD13 UBE2V1 KCTD6 SOCS3 ASB7 FBXO21 RLIM KLHL11 UNKL ASB16 UBE2Q1 SH3RF1 ASB6 FBXL20 CBLB RNF138 UBE2D4 ANAPC1 ATG7 BTRC PTGER3 GPR37L1 CORT C5AR1 GNAI2 CCR1 GNG3 ADCY9 C5 ADCY2 CXCR6 CXCL12 CCL4L1 CCL4 APLN SST KCTD7 CNR1 ARRB1 |
| hsa_circRNA_103831 | hsa-miR-625-3p  hsa-miR-129-5p  hsa-miR-548c-3p  hsa-let-7c-5p  hsa-let-7b-5p | FBXO32 GAN KLHL13 LTN1 RNF111 HECTD2 RNF217 UBE2G2 ZNRF1 RCHY1 CBLB HACE1 FBXO30 CDC34 ATG7 SKP1 SOCS1 TRIM41 NEDD4L FBXW7 TRIM71 SIAH1 TP53 CCND1 AGO1 TNRC6A AGO4 AGO3 TNRC6C TNRC6B EZH2 CCND2 EP300 DICER1 CDK6 E2F2 WNT5A SYT1 SYT2 SYT11 VAMP4 FZD4 ADRB2 SH3KBP1 STON2 FCHO2 AAK1 HIP1 CASP3 MAPK1 |
| hsa_circRNA_103828 | hsa-miR-411-5p  hsa-miR-625-3p  hsa-miR-448  hsa-miR-205-5p  hsa-miR-526b-5p | UBE2Z HECTD2 HERC3 UBE2W CDC27 RNF217 RNF19A GAN RNF111 PJA2 RNF4 KLHL5 ZNRF2 RCHY1 FBXO22 UNKL DTX3L UBE2Q1 FBXL20 MGRN1 UBE2K ASB7 WWP1 HECW2 SIAH1 NEDD4L SMURF1 MYLIP CTTN SH3KBP1 SGIP1 NECAP1 SH3GL3 STAM AAK1 PICALM SYT9 STON2 KIAA0319 CKAP4 CSF1 PDIA6 LAMC1 CALU SCG2 RCN1 KTN1 ANO8 MGAT4A RANBP2 |
| hsa_circRNA_103752 | hsa-miR-141-3p  hsa-miR-29a-5p  hsa-miR-499a-3p  hsa-miR-570-5p  hsa-miR-26a-5p | HECTD1 RNF41 UBE2E3 RNF6 SIAH1 UBE2F RCHY1 DTX3L SH3RF1 FBXL20 KEAP1 FBXW2 CDC27 UBA6 UBE2E1 UBE3A FBXO11 KLHL42 CUL3 ITCH UBE2J1 UBE2W UBE2G1 UBE2H UBE2D3 UBE2D1 UBR1 FBXW7 CBLB ASB7 SRSF1 SRSF11 SRSF2 CDC40 SF3B1 HNRNPA2B1 HNRNPU HSPA8 SNRNP40 PRPF40A CRNKL1 CWC27 FIP1L1 CPSF2 RBM5 ELAVL2 DCUN1D1 COMMD8 TCERG1 DCUN1D3 |
| hsa_circRNA_071106 | hsa-miR-1206  hsa-miR-29a-5p  hsa-miR-6830-3p  hsa-miR-4743-3p  hsa-miR-2682-3p | WDR33 CPSF2 PPIL6 SNRNP40 WBP11 CWC27 GPSM3 U2SURP HNRNPR HNRNPH1 HNRNPU CDC40 SART1 TXNL4A RNF41 TRIM39 RNF217 KBTBD6 RNF213 KLHL5 RNF34 KLHL9 LONRF1 UBE2V1 FBXO40 UBE2V2 UBE2F KLHL11 UNKL DTX3L UBOX5 PRPF38A WWP1 UBA6 UBE2E1 CCAR1 VHL UBE3A SF3B3 ASB7 TRIP12 ITCH UBE2W UBE2H UBE2G2 UBE2B DDX46 NCBP2 SIAH1 PTGER3 |
| hsa_circRNA_102293 | hsa-miR-340-3p  hsa-miR-30a-5p  hsa-miR-145-5p  hsa-miR-30b-5p  hsa-miR-30e-5p | SOCS6 TULP4 CAND1 DCUN1D1 UBE2D3 WWP1 UBE2W HECTD1 CUL2 NEDD4 HERC3 UBE2F FBXO32 FBXL3 FBXL14 UBE2G2 FBXO40 ZNRF1 RLIM KLHL11 UBA6 SPSB4 UNKL TRIM9 DTX3L SH3RF1 FBXL20 UBE2R2 HACE1 HERC2 HECW2 VHL KLHL20 AREL1 CUL5 TRIP12 DCUN1D3 SKP1 SKP2 KCTD7 NEDD4L CBLB UBA52 RAB5C RAB5B AGFG1 EPS15 IGF2R CFTR ITSN1 |
