# Supplementary material for: Potential and functional prediction of six circular RNAs as diagnostic markers for colorectal cancer
Source: PeerJ. 2022 May 19;10:e13420. doi: 10.7717/peerj.13420 (PMC9124462; doi:10.7717/peerj.13420)
Supplement: Supplemental Information 7 [file peerj-10-13420-s007.docx]

**Miame Checklist**

Part 1 Experiment description

-         **experiment type: human**

-         **experimental variables (CRC tumor vs. paracancerous tissues)**

**-         n-count: 3 vs 3**

-         **tissues used for slide : CRC tumor**

-         **mouse age, and other variables :** the mean age of the 3 patients used for microarray is 63 years

Part 2 Array design.

-         **Array series** circRNA Microarray Service for 6 human samples

-         **Deconvoluted spot list with gene names** The microarray data are available through the GEO database with accession number GSE156719.

-         **Array type (mouse, human, cDNA, oligo, number of genes)**Human circRNA microarray

-         **Array size**

-         **Slide type (and coating)**

Part 3 Samples

-         **Cy3/Cy5 labels for tissues** Cyanine-3 (Cy3)

-         **Dye swap? Or reference control?**

-         **Labelling protocol used** Sample labeling and array hybridization were performed according to the manufacturer’s protocol (Arraystar Inc.). Briefly, total RNAs were digested with Rnase R (Epicentre, Inc.) to remove linear RNAs and enrich circular RNAs. Then, the enriched circular RNAs were amplified and transcribed into fluorescent cRNA utilizing a random priming method (Arraystar Super RNA Labeling Kit; Arraystar).

-         **Sample extraction protocol used** total RNAs were digested with Rnase R (Epicentre, Inc.) to remove linear RNAs and enrich circular RNAs.

-         **Amount of sample labelled** 6 human samples

Part 4 Hybridizations

-         **Hybridization protocol** The labeled cRNAs were purified by RNeasy Mini Kit (Qiagen). The concentration and specific activity of the labeled cRNAs (pmol Cy3/μg cRNA) were measured by NanoDrop ND-1000. 1 μg of each labeled cRNA was fragmented by adding 5 μl 10 × Blocking Agent and 1 μl of 25 × Fragmentation Buffer, then heated the mixture at 60 °C for 30 min, finally 25 μl 2 × Hybridization buffer was added to dilute the labeled cRNA. 50 μl of hybridization solution was dispensed into the gasket slide and assembled to the circRNA expression microarray slide. The slides were incubated for 17 hours at 65°C in an Agilent Hybridization Oven. The hybridized arrays were washed, fixed and scanned using the Agilent Scanner G2505C.

-         **ALL modifications and deviations from the protocol**

-         **Manual hybridization or automatic chamber?**

-         **Number of slides done at the same time**

-         **Hyb time**

-         **Number of washes**

-         **Amount of labelled sample hybridized** 6

-         **Labelling efficiency**

Labeling Efficiency-QC

In the experiment, 1μg RNA is used for labeling. The specific activity (pmol dyes per μg cRNA) of the labeled RNA can be obtained by the following calculation:

(pmol per μl dye)
Specific Activity =——————————
 (μg per μl cRNA)

*For two-color, if the yield is <825 ng and the specific activity is <8.0 pmol Cy3 or Cy5 per μg cRNA do not proceed to the hybridization step. Repeat cRNA preparation.
*For one-color, if the yield is <1.65μg and the specific activity is <9.0 pmol Cy3 or Cy5 per μg cRNA do not proceed to the hybridization step. Repeat cRNA preparation.

Part 5 Measurements

-         **Which version of scanner software used** Agilent Feature Extraction software (version 11.0.1.1)

-         **Laser power for scan**

-         **Instrument model numbers**  Agilent Scanner G2505C

-         **Must save original .tiff format images (composite image is optional)**

*For each experimental image, a microarray quantification matrix contains the complete image analysis output as directly generated by the image analysis software (normally provided as separate spreadsheet-type files). Note that for a given image this is a 2D matrix, where array elements (spots or features) constitute one dimension and quantification types (such as mean and median intensity, mean or median background intensity) are the second dimension.*

-         **Normalization protocol**

-         **Does the scanner software subtract background? How much?**

-         **Spot raw values, background intensity, ch1 and 2 intensity, etc.**

-         **Corresponding gene name**

-         **Methods of analysis (MAN, Spotfire, Genespring) be detailed.**

-         **Normalized to controls? Controls removed? All normalization parameters**

-         **Name of Images, Experiment, and location of files.**

-         **Lowess or other normalization if used (and parameters)**

*Finally, the gene expression matrix (summarized information) consists of sets of gene expression levels for each sample. If microarray quantification matrices can be considered spot/image centric, then the gene expression matrix is gene/sample centric. At this point, the expression values may have been normalized, consolidated and transformed in any number of ways by the submitter in order to present the data in a form amenable to scientific analysis. Rather than attempting to impose a standard for gene expression values, MIAME indicates preferred detailed specifications of all numerical calculations applied to unprocessed quantifications in (b) that have led to the data in (c). Experimenters are encouraged, though not required, to provide reliability indicators (such as s.d.) for each data point.*

-         **Output file**

-         **Normalized ratios**

-         **Numerical manipulations**

-         **Cut off values**

Part 6 Normalization controls

*“A typical microarray experiment involves a number of hybridization assays in which the data from multiple samples are analyzed to identify relative changes in expression levels, identify differentially expressed genes and, in many cases, discover classes of genes or samples having similar patterns of expression.”*

-         **Hypothesis**

-         **Gene expression patterns found**

-         **Controls used, normalization methods used (see above)**
